# Leader cells mechanically respond to aligned collagen architecture to direct collective migration

**DOI:** 10.1371/journal.pone.0296153

**Published:** 2024-01-02

**Authors:** Jessanne Y. Lichtenberg, Ella Ramamurthy, Anna D. Young, Trey P. Redman, Corinne E. Leonard, Swadesh K. Das, Paul B. Fisher, Christopher A. Lemmon, Priscilla Y. Hwang

**Affiliations:** 1 Department of Biomedical Engineering, Virginia Commonwealth University, Richmond, Virginia, United States of America; 2 Department of Bioengineering, University of California Berkeley, Berkeley, California, United States of America; 3 Department of Human and Molecular Genetics, School of Medicine, Virginia Commonwealth University, Richmond, Virginia, United States of America; 4 VCU Institute of Molecular Medicine, School of Medicine, Virginia Commonwealth University, Richmond, Virginia, United States of America; 5 VCU Massey Cancer Center, School of Medicine, Virginia Commonwealth University, Richmond, Virginia, United States of America; University of Bayreuth, GERMANY

## Abstract

Leader cells direct collective migration through sensing cues in their microenvironment to determine migration direction. The mechanism by which leader cells sense the mechanical cue of organized matrix architecture culminating in a mechanical response is not well defined. In this study, we investigated the effect of organized collagen matrix fibers on leader cell mechanics and demonstrate that leader cells protrude along aligned fibers resulting in an elongated phenotype of the entire cluster. Further, leader cells show increased mechanical interactions with their nearby matrix compared to follower cells, as evidenced by increased traction forces, increased and larger focal adhesions, and increased expression of integrin-α2. Together our results demonstrate changes in mechanical matrix cues drives changes in leader cell mechanoresponse that is required for directional collective migration. Our findings provide new insights into two fundamental components of carcinogenesis, namely invasion and metastasis.

## Introduction

Collective migration, the movement of clusters of cells together, is one of the main modes of migration by which tumor cells invade or metastasize in vivo [1–4]. During collective migration, cancer cells migrate in a cohesive, multicellular unit [1, 5–8] and are led by a subgroup of cells, known as leader cells, located at the front edge. Work in our lab and others have found that leader cells are positive for cytokeratin-14 (K14) in collective migration of breast cancer cells [1, 9, 10]. Further, our lab has observed K14+ leader cells are located throughout tumor clusters but in order for directional collective migration to occur, K14+ leader cells must polarize to the front edge to lead collective migration with associated collagen fiber alignment and thickening in the direction of migration [10]. In tumor clusters that did not migrate, collagen fibers remained curly and disorganized [10]. While these findings correlate with *in vivo* observations comparing metastatic tumors with non-metastatic tumors [11, 12], the relationship between matrix architecture and leader cell sensing or activation required for collective migration is still not fully understood.

Extracellular matrix (ECM) properties are believed to contribute to cell migration and associated mechanosignaling in tumor cells thereby effecting disease progression. In a murine model of breast cancer, individual tumor cells migrate along collagen fibers, suggesting collagen fibers provide a track or roadmap for cells to traverse [13]. Additionally, cells exert mechanical traction forces on their microenvironment during migration with associated focal adhesion organization and actomyosin contractility [13, 14]. Focal adhesions increase their strength in response to force and trigger protrusions of the cell membrane [15], such as finger-like filopodia and ruffling lamellipodia [16]. During collective migration, leader cells also send out protrusions [17], but the correlation to force exertion and changes in focal adhesion organization is still largely unknown.

When focal adhesions are formed, vinculin, a known regulator of cell directed migration and traction force generation, is recruited from the adherens junctions to focal adhesion sites [15, 18–20]. When vinculin is recruited to focal adhesions, focal adhesions are stabilized and integrins cluster to form complexes that can engage with the ECM [15, 21]. One integrin that has been identified as a key regulator of adhesion, motility, and invasiveness in cancer cells is integrin-α2 (ITGα2) [22, 23]. Further knowledge regarding the relationship between traction forces, focal adhesions and integrin-mediated signaling in leader cells are needed to understand the role of mechanical cues and forces in leader cells that regulate collective migration.

Here we demonstrate that leader cells are mechanically active and sense the tumor microenvironment through exerting focal adhesion mediated traction forces. Using custom generated 3D aligned and random collagen hydrogels, we demonstrate leader cells polarize along collagen fibers to form protrusions that aid in cell spreading. Further, measurement of cell exerted traction forces and quantification of vinculin focal adhesions demonstrate leader cells generate greater traction forces with more vinculin focal adhesions compared to follower cells. This suggests that leader cells are more mechanically active than follower cells, which is essential for initiating collective migration. Finally, we observe higher expression of ITGα2 in leader cells compared to follower cells, which may imply leader cells use ITGα2 as part of their mechanosensing machinery to sense mechanical environment cues and drive migration. Our findings reveal a mechanosensing role in leader cells and provide a springboard for future studies to probe downstream signaling pathways underlying this phenomenon. Understanding how leader cells mechanically respond during collective migration would help inform mechanosignaling pathways that could serve as targets in therapy development to prevent metastasis.

## Results

### Leader cells polarize along aligned fibers resulting in elongated tumor organoid morphologies

**Fig 1:**
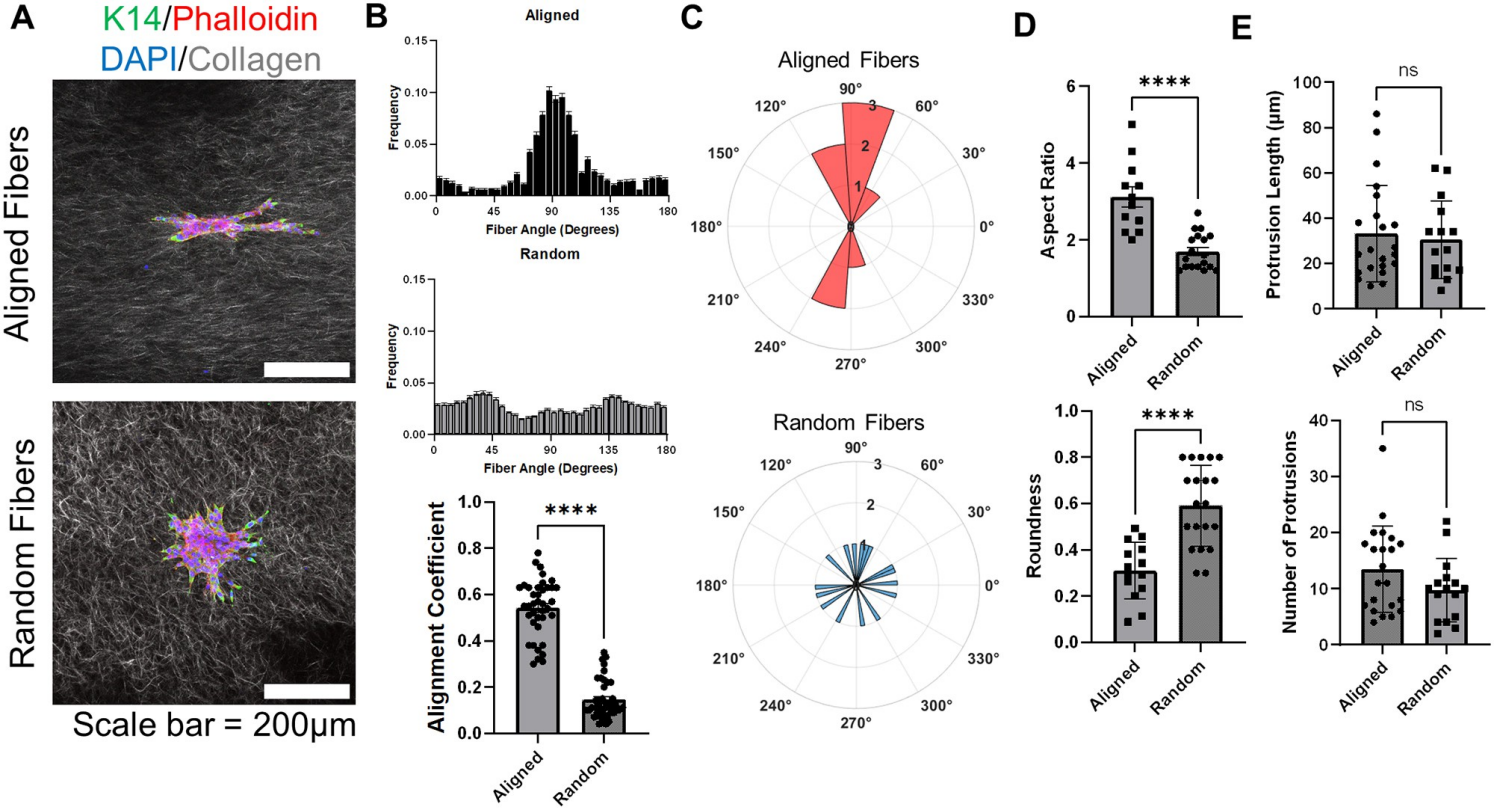
Leader cells polarize and protrude along aligned fibers resulting in elongated tumor organoids. (A) Representative immunofluorescence images of tumor organoids in aligned and random collagen fibers (confocal reflectance) (K14 = green, phalloidin = red, DAPI = blue, collagen = white). Scale bar = 200 μm. (B) Fiber angle frequency and alignment coefficient of collagen fiber orientation in aligned and random hydrogels. n = 39–40 per condition. (C) Representative rose plots of organoid protrusion angles in aligned or random collagen matrices. (D) Organoid morphology characterization: roundness (1 indicates perfectly round), and aspect ratio (higher value indicates more elongated shape) (E) Quantification of organoid protrusions for length and number of protrusions. n = 36 organoids from three different mice. All data shown as mean ± SEM. ns indicates p>0.05, ****p<0.0001, with an unpaired t-test.

Since prior studies indicate individual tumor cells respond to collagen fiber alignment [12, 13], we wanted to understand if collagen fiber alignment could affect collective migration. We generated 3D aligned and random collagen hydrogels [24, 25] using magnetic beads, and cultured primary tumor organoids. First, we validated the generation of aligned and random fibers in our collagen hydrogels (Fig 1A and 1B). After culturing primary tumor organoids in the aligned and random hydrogels, we observed that primary tumor organoids spread out in an elongated manner in the direction of aligned collagen fibers, whereas tumor organoids spread radially in random collagen fibers (Fig 1C). Further quantification of organoid characteristics indicate organoids are more elongated (roundness, aspect ratio) in aligned fibers compared to random fibers (Fig 1D). Finally, we quantified the length and number of protrusions in leader cells (Fig 1E) and did not observe significant differences in the length and number of protrusions on the organoids between the aligned and random fiber hydrogels. At the tip of all protrusions, we observed K14+ leader cells (Fig 1A) at the front, suggesting leader cells respond to fiber alignment cues that result in elongated bulk morphologies of tumor organoids. This suggests that the fiber orientation mainly affects the direction of the protrusions rather than the number or length.

### Leader cells exert increased traction forces through focal adhesions

**Fig 2:**
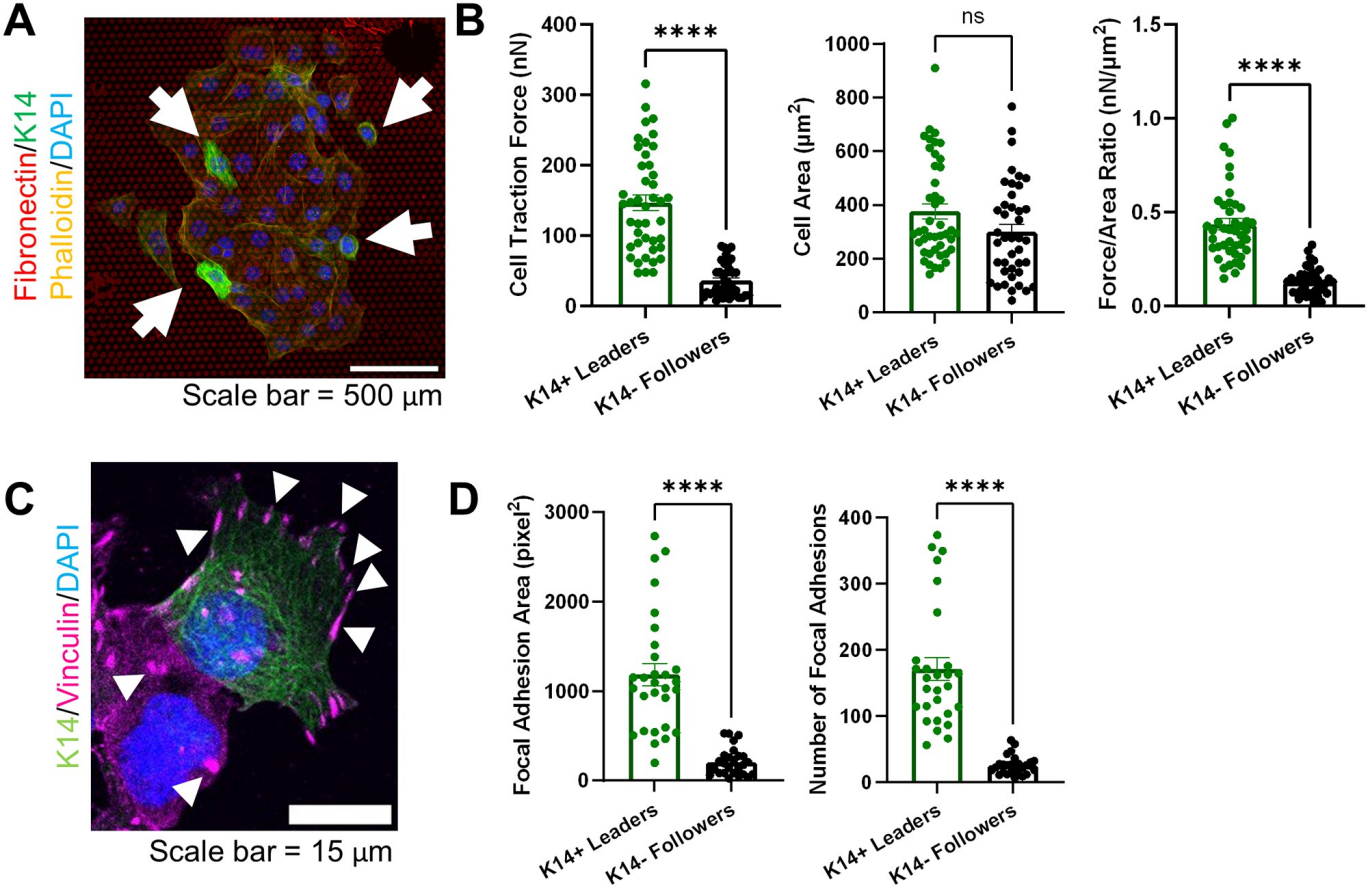
K14+ leader cells in tumor organoids generate increased traction forces and vinculin focal adhesions in 2D. (A) Representative immunofluorescent image of tumor organoid on protein-patterned dot grid for 2D traction force measurement. (HexForce dots = red, K14 = green, Phalloidin = yellow, DAPI = blue) Scale bar = 500μm. White arrows highlight K14+ leader cells. (B) Analysis of cell traction forces exerted, cell area, and force/area ratio. n = 42 organoids from three mice. (C) Representative immunofluorescent image of K14+ leader and K14- follower cells for K14 (green) and vinculin (pink) with nuclei counterstain, DAPI (blue). White arrowheads highlight vinculin focal adhesions (pink). Scale bar = 15 μm. (D) Quantification for the number of focal adhesions and area in K14+ leader cells and K14- follower cells. n = 24 organoids from three mice. All data shown as mean ± SEM. ns indicates p>0.05, ****p<0.0001, with an unpaired T-test.

To study how leader cells interact with their surrounding matrix mechanically, we measured the traction forces of tumor organoids using a HexForce assay [26] (Fig 2A). We cultured primary tumor organoids on HexForce substrates and took images of the entire organoid after staining for phalloidin (counterstain DAPI). Since leader cells are genetically marked with GFP, during our analysis, we visually identified K14+ leader cells and K14- follower cells, and quantified traction forces of these two populations. We found that K14+ leader cells generated greater traction forces compared to K14- follower cells (Fig 2B). Both leader and follower cells had similar cell areas, suggesting that the increased forces by the leader cells was not due to size differences but rather increased mechanical engagement with the ECM (Fig 2B). Since cell-ECM interactions are largely regulated by focal adhesions, we determined if focal adhesions were different between leader and follower cells; thus we stained tumor organoids for vinculin, a known focal adhesion protein regulating migration [18–20]. K14+ leader cells had more and larger focal adhesions than K14- follower cells (Fig 2D). Vinculin recruitment at the cell-matrix interface and specifically at the cell edges is indicative of cell spreading and increased force exertion [27]. In fact, we observed more focal adhesions at the cell edges in leader cells compared to follower cells (Fig 2C). Finally, we observed that focal adhesions on K14+ leader cells are localized at the cell-matrix intersections and cellular protrusions, which correlates with other studies that show focal adhesions localize at the leading edge of cells [28]. Overall, these findings indicate K14+ leader cells are more mechanically engaged with the ECM than K14- follower cells.

### K14+ leader cells express increased integrin-α2 (ITGα2) compared to K14- follower cells

**Fig 3:**
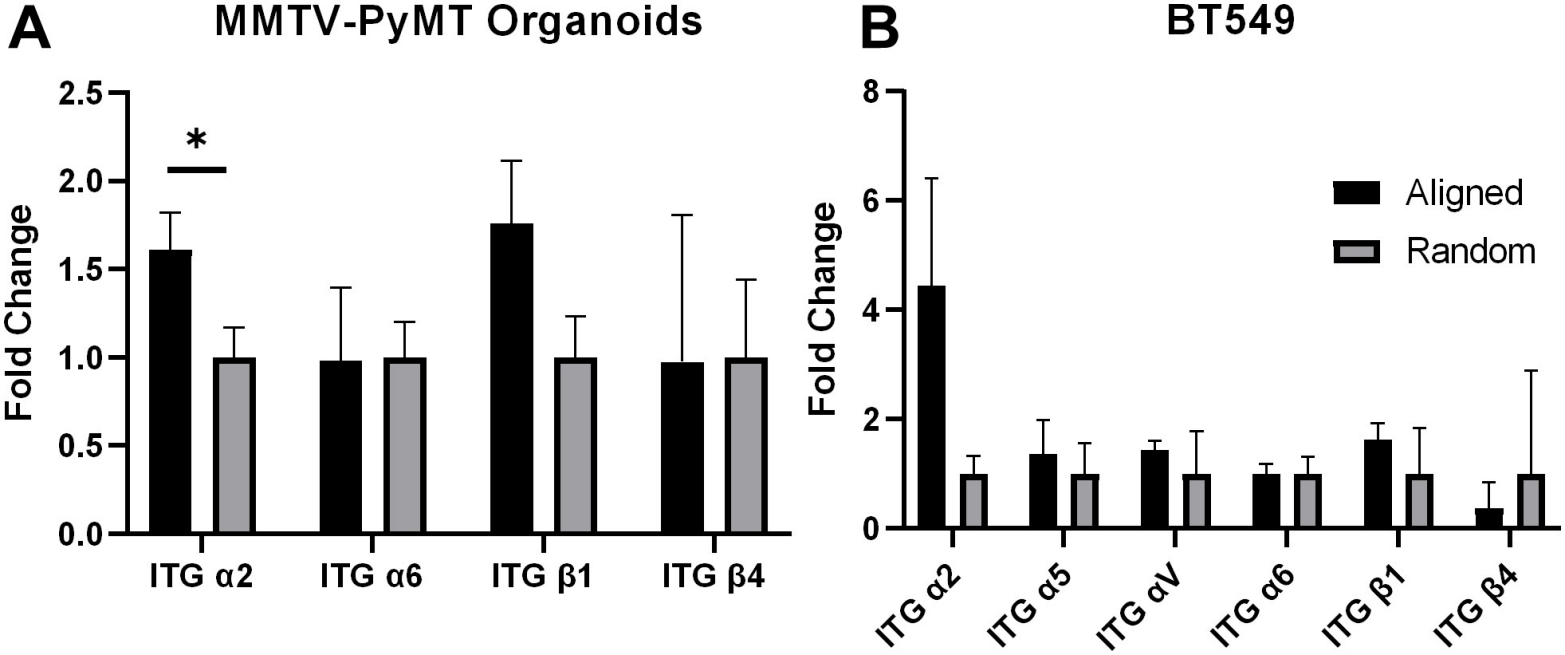
Leader cells have increased integrin-α2 expression compared to follower cells. Integrin gene expression panel from RNA extracted from (A) tumor organoids (n = 3 independent mouse models) and (B) BT549 (K14+ human breast cancer cell line) cells in aligned and random fibers. *p<0.05, with an unpaired T-test.

**Fig 4:**
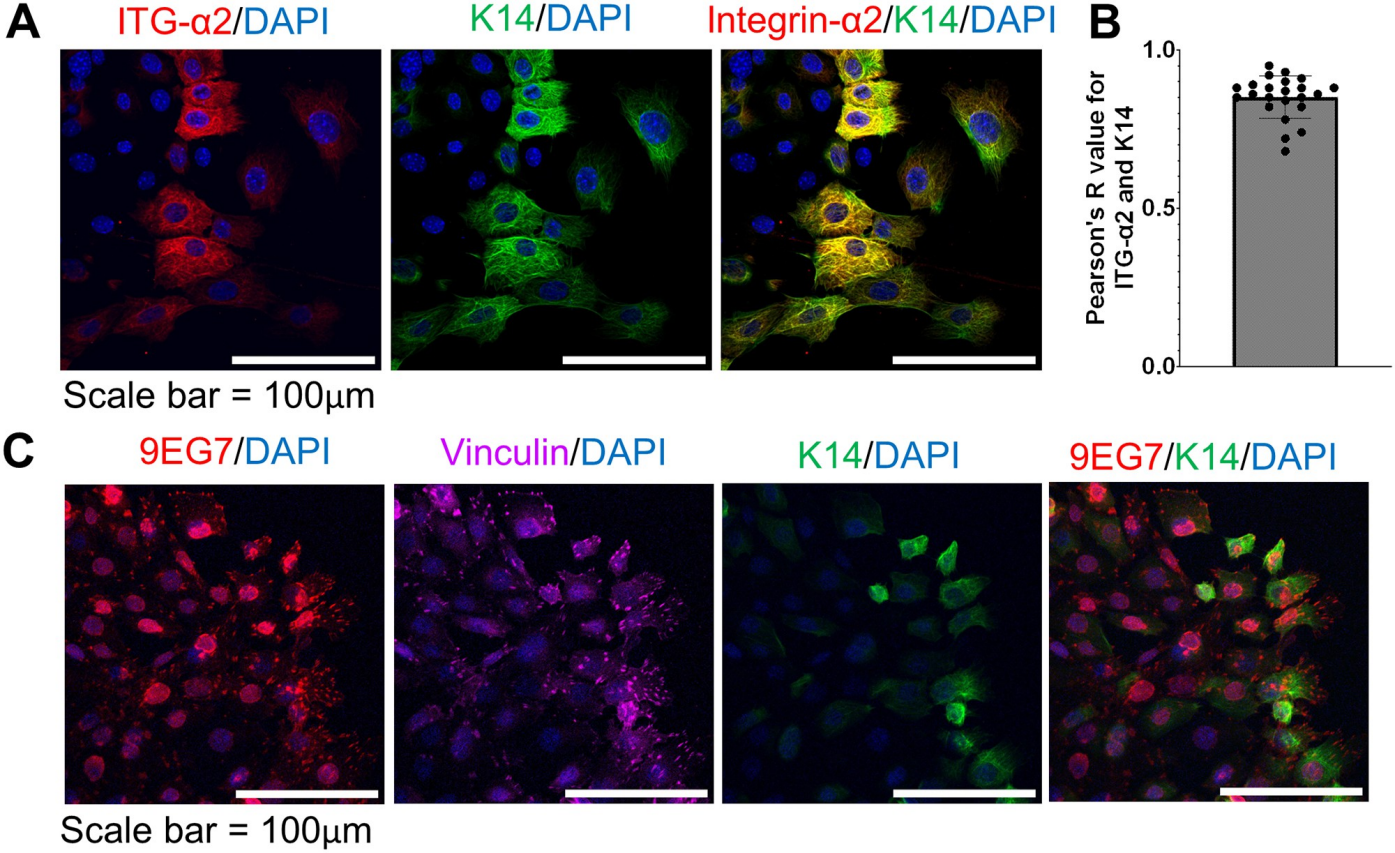
Integrin-α2 colocalizes with K14 in leader cells. (A) Representative immunofluorescent images of tumor organoids with integrin-α2 (red), K14 (green) and nuclei (blue). (B) Pearson’s R value from colocalization analysis of integrin-α2 and K1. n = 23 organoids from three mice. Data shown as mean ± SEM. (C) Representative immunofluorescent images of tumor organoids with activated integrin-β1 (9EG7, red), vinculin (pink), K14 (green) and nuclei (blue).

Since focal adhesions connect the cell cytoskeleton with integrins that are used to interact with the extracellular matrix, we wanted to understand which integrins are critical for matrix architecture sensing. We performed qRT-PCR on a panel of integrins known to regulate cell migration after tumor organoids spread on 3D aligned and random collagen hydrogels (Fig 3A). Our qRT-PCR results reveal statistically significant increased gene expression of ITGα2 in organoids cultured on aligned collagen hydrogels compared to random collagen hydrogels (Fig 3A). There was also observed increase in gene expression of ITGB1, however it was not statistically significant. To further understand which integrins may regulate matrix architecture sensing in leader cells specifically, we also cultured BT549 human tumor cells on aligned and random collagen hydrogels. BT549 cells are a representative model for leader cells as they are positive for K14 [29]. qRT-PCR demonstrates that BT549 cells have increased expression of ITGα2 on aligned collagen compared to random collagen (Fig 3B). To further confirm our gene expression results, we stained the organoids for ITGα2, activated ITGβ1 (9EG7), vinculin, and integrin-β1 on collagen 1/fibronectin coated PDMS-spun coverslips (Fig 4A and 4B). We observed positive expression of ITGα2in the K14+ leader cells, but not the K14- follower cells (Fig 4A). This was confirmed through colocalization analysis which revealed a strong positive correlation between ITGα2 and K14 staining intensities (Fig 4A). ITGβ1 was ubiquitous on all cells (S1 Fig), but there appears to be a moderate positive correlation between activated ITGβ1 and vinculin staining (Fig 4B and 4D), which supports previous findings that increased ITGβ1 activation is associated with increased traction forces [30]. Similarly to vinculin, we observed the presence of activated integrin-B1 is greater in the leading edges of K14+ leader cells and greater in K14+ leader cells than K14- follower cells, which is consistent with previous studies which showed ITGβ1 was highly localized in the lamellipodia of leader cells with enhanced presence in leader cells than in follower cells [31]. Altogether, these results begin to suggest that activated ITGβ1, ITGα2, and vinculin may have a unique function in K14+ leader cells correlated with increased force exertion by leader cells.

## Discussion

This study sought to better understand leader cell mechanics during collective migration in response to aligned collagen architecture. Our findings demonstrate breast tumor leader cells sense collagen fiber orientation and use it to preferentially align in the direction of the fibers. Further, we found leader cells exert increased traction forces and form larger and more focal adhesion complexes compared to follower cells. These differences are accompanied by a colocalization of ITGα2 with K14, a known leader cell marker in breast cancer.

While much work has demonstrated single cells migrate along the path of aligned collagen [13], how aligned collagen architecture effects collective migration was not investigated. Present findings correlate with other studies that demonstrate leader cells respond to matrix fiber alignment during wound healing [32], an example of 2D collective migration. The phenomenon of collective cells migrating differently based on collagen architecture is supported by a recent study that saw differences in tumor spheroid and organoid migration in radially versus circumferentially aligned fibers [33]. Other studies have demonstrated cells migrate faster and more efficiently along an aligned matrix [14]. In addition to increased migration speed and efficiency, cell migration in 3D aligned fibers has been shown to have increased persistence through protrusion dynamics and focal adhesions arrangement and shape [13]. Additionally, the alignment of collagen fibers produces a stiffer matrix than random fibers which could also contribute to cell persistence [34].

Our study demonstrates leader cells exert higher traction forces with associated larger and greater number of vinculin focal adhesions compared to follower cells. These findings suggest leader cells are mechanically responsive in a manner that follower cells are not. Correlation between the amount of traction force generated and large vinculin focal adhesions have been investigated in single cells [18], but less so for groups of cells that are migrating together. During active single cell migration, vinculin localizes at the front of the cell and spread out in a particular direction, whereas vinculin localization is not observed in cells that invade without directionality [15, 28]. These findings, along with current findings that leader cells display prominent vinculin focal adhesions that are oriented along a cell’s leading edge, further point towards a unique mechanical role in leader cells that is required for directional collective migration.

Characterization of integrin expression of tumor clusters cultured on aligned versus random hydrogels reveal higher expression of ITGα2 in aligned collagen. Further, immunostaining of ITGα2 in tumor organoids demonstrate leader cells have positive expression of ITGα2. Our observation correlates with other published studies that also demonstrate unique gene and protein expression of ITGα2 in leader cells that is absent in follower cells via genetic analysis [16]. ITGα2 is a key regulator of adhesion in cancer cells [22], and blockage of ITGα2 in murine models of breast cancer have resulted in reduced metastasis to the liver [35]. ITGα2, when bound with ITGβ1 in mammalian cells senses the extracellular matrix that it is bound and translates that specific signal to activate intracellular signaling pathways [22, 35]. Inside the cell, integrins are linked to actin filaments through focal adhesion proteins, thus affecting cell contractility through the focal adhesion kinase (FAK) pathway [36]. Since focal adhesions are being constantly assembled and disassembled as cells migrate, future work includes investigating focal adhesion dynamics to see if changes in matrix affects leader cells’ ability to form and maintain focal adhesions.

As a whole, this work demonstrates leader cells polarize in the direction of fiber orientation and leader cells are more mechanically active than follower cells. Additionally, this study starts to investigate the role of vinculin in the mechanical activity of leader cells. Our findings reveal the importance of leader cell mechanics that contributes to metastasis and begin to probe a mechanism of how leader cells mechanically engage with the ECM. Understanding how leader cells respond to mechanical cues will lead to future studies of the downstream signaling pathways involved creating a comprehensive understanding of collective migration and metastasis. This information could inform the design of target specific therapeutics to block and potentially prevent cancer cell invasion and metastasis.

## Materials and methods

### 3D collagen invasion assay

Collagen 1 hydrogels (3 mg/ml) were made from rat tail collagen 1 (Corning, 354236), sodium hydroxide (1M, Sigma-Aldrich 72068-100ML), 10X DMEM low glucose (Sigma-Aldrich, D2429), and 10X PBS (Fisher Scientific BP3994), adapted from [24]. Neutralized collagen underlays were loaded into a 4 well chamber slides (Lab-Tek 155383). Aligned underlays were created by incorporating 1% paramagnetic beads (Spherotech PM2010) into the collagen hydrogel solution and polymerizing directly adjacent to a magnet (K&J Magnetics BY084-N52) for 30 min at 37°C (25). Random collagen underlays had no paramagnetic beads and were polymerized under the same conditions. Tumor organoids were added onto the overlays (about 100 organoids per well) and allowed to adhere for 30 min at 37°C. Random collagen overlays were added on top of all the gels and incubated for 30 min. Media (DMEM-F12 (11330032, Gibco), 1% penicillin-streptomycin (Corning, MT30002CI), 1% insulin-transferrin-selenium-ethanolamine (41400045, Gibco), 2.5 nM FGF2 (F0291, Sigma)) was added to the wells and organoids were incubated for 2–4 days at 37°C until invasive. Cells were fixed in 4% PFA and blocked in 1X PBS with 1% BSA and 0.1% Tween20. Organoids were washed in PBS with 1% Tween20 and stained for cytokeratin-14 (Biolegend 905301, 1:500x), and Alexa Fluor^™^ 568 Phalloidin (Invitrogen A12380, 1:500x) overnight at 4°C. A secondary antibody, goat anti-rabbit Alexa Fluor^™^ 488 (Invitrogen A-11008, 1:500x), and nuclei staining (DAPI) were also used. Organoids were imaged via confocal microscopy (Zeiss LSM 710 or 980) at 20x air 1.0 zoom with slices taken every 10 μm for the z-stack. The collagen fibers were visualized using confocal reflectance microscopy with a 405 nm laser. Images were adjusted for brightness and contrast in FIJI/ImageJ. Confocal reflectance images were analyzed for collagen fiber orientation using CURVE Align software [37–40]. The fiber orientation histogram generated in CURVE Align is based on the frequency of each fiber angle in degrees within an image. To calculate the alignment coefficient, each fiber angle is multiplied by 2 to map to the orientation range of [0–2π], and then the alignment coefficient is calculated as the normalized vector sum of orientation vectors or the mean resultant vector length in circular statistics [37]. The alignment coefficient ranges from 0 to 1, where 0 indicates perfectly random fibers and 1 indicates perfectly aligned fibers. To generate the polar plots of the protrusion angles of the organoids, we used the actin immunofluorescent staining images to create an outline of the organoid perimeter in FIJI. The centroid of each organoid was found using the Analyze Particles tool. Each protrusion was measured starting from the centroid for the angle and length. The angles were converted from degrees to radians in MATLAB and the polar histograms were created. Analysis of organoid morphology was performed in FIJI using Analyze Particles shape descriptors tool. Roundness ranges from 0 to 1 where 1 indicates a perfectly round shape. Aspect ratio indicates elongation, where a higher aspect ratio indicates greater elongation.

### 2D traction force measurement

Traction forces were measured using the HexForce method [26]. Briefly, blank PDMS stamps (1cm x 1cm x 0.5 cm, made from 1:10 Sylgard 184) were coated in a solution of rhodamine fibronectin (Cytoskeleton FNR01) and rat tail collagen 1 (Cultrex) at a 1:20 dilution (50 μg/ml). UV-treated negative stamps and PDMS-coated glass coverslips (25mm diameter, 3kPa stiffness) were brought into conformal contact with the protein coated blank stamp. Patterned coverslips were incubated in F-127 Pluronic blocking solution (1% in PBS) before cell seeding. Tumor organoids were prepared as described in the mouse model section and seeded at a density of 1000 organoids per sample. Cells were incubated for 16hr at 37°C. The samples were fixed in 4% PFA and blocked in 1X PBS with 0.1% BSA. Organoids were stained for Alexa Fluor^™^ Plus 647 Phalloidin (Invitrogen, 1:500x), cytokeratin-14 (Biolegend 905301, 1:500x), and vinculin (Sigma Aldrich V9131, 1:500x). Secondary antibodies include goat anti-rabbit Alexa Fluor^™^ 488 (Invitrogen A-11008, 1:500x) and donkey anti-mouse Alexa Fluor^™^ 568 (Invitrogen, 1:500x). Cell nuclei were stained for DAPI. The tumor organoids were imaged on a Nikon Ti2 using 60x air objective. Fluorescent images of K14 (leader cell marker) were subtracted from the phalloidin or vinculin images of whole tumor organoids to produce an image of just the follower cell phalloidin or vinculin. These separated images were used in the characterization of traction forces and focal adhesions. Traction forces of leader cells and follower cells were quantified using a custom MATLAB code [26]. All HexForce MATLAB analysis code is available at www.lemmonlab.com. To visualize vinculin, the samples were imaged via confocal microscopy (Zeiss LSM 980) at 63x air with slices taken every 1 μm for a z-stack. Vinculin was analyzed with the Focal Adhesion Analysis Server [41].

### 2D immunofluorescence staining

Blank PDMS stamps (1cm x 1cm x 0.5 cm, made from 1:10 Sylgard 184) were coated in a solution of fibronectin (Sigma-Alrich, F1141) and rat tail collagen 1 (Cultrex) at a 1:20 dilution (50 μg/ml). UV-treated PDMS-coated glass coverslips (25-mm diameter, 3kPa stiffness) were brought into conformal contact with the protein coated blank stamp. Protein coated coverslips were incubated in F-127 Pluronic blocking solution (Sigma-Aldrich P2443, 1% in 1X PBS) before cell seeding. Tumor organoids were prepared as described in the mouse model section and seeded at a density of 1000 organoids per sample. Cells were fixed in 4% PFA and blocked in 1X PBS with 1% BSA and 0.1% Tween20. Cells were washed in PBS with 1% Tween20. Primary antibodies include cytokeratin-14 (Biolegend 905301, 1:500x), vinculin (Sigma Aldrich V9131, 1:500x), integrin-β1 (Cell signaling technologies 4706, 1:500x), ITGα2 (Invitrogen PA5-47193, 1:500x) and activated integrin-β1 (9EG7) (BD Biosciences 553715, 1:50x). Secondary antibodies include goat anti-mouse Alexa Fluor^™^ 647 (Invitrogen, 1:500x), goat anti-rabbit Alexa Fluor^™^ 488 (Invitrogen, 1:500x), goat anti-rat Alexa Fluor^™^ 594 (Invitrogen, 1:500x), and goat anti-rabbit Alexa Fluor^™^ 555 (Invitrogen, 1:500x). Organoids were counterstained for DAPI. Organoids were imaged via confocal microscopy (Zeiss LSM 980) at 63x air 1.0 zoom with slices taken every 1 μm for a z-stack. Colocalization was performed using FIJI Coloc 2.

### Tumor organoid preparation

As a primary source of cells, we isolate clusters of primary tumor cells, known as organoids, from a spontaneous breast cancer mouse model (MMTV-PyMT) because tumor cells from this model are established to migrate collectively [1, 7, 9, 10]. Mammary gland tumors were isolated from female 12 week old MMTV-PyMT;K14-GFP mice gifted from the Longmore lab [10, 24], following the approved IACUC protocol (AD10002197). The mice were monitored weekly and euthanized at 12 weeks, or if mice showed adverse effects. Euthanasia was performed and confirmed by cervical dislocation. Freshly isolated tumors were minced and digested in low concentrations of collagenase and Trypsin as previously published [10]. Tumor organoids were separated out by differential centrifugation and filtered to be between 40–100 μm [24]. Tumor organoids were used immediately for all experiments to avoid culturing on tissue culture plastic. All organoids are cultured in standard culture media (DMEM 1X supplemented with 10% HI FBS and 1% Penicillin-streptomycin).

### Gene expression

Tumor organoids or human breast cancer cell line BT549 (ATCC) in 3D aligned or random collagen gels were cultured for 3–6 days until invasive at 37°C. Cells were cultured in DMEM 1X supplemented with 10% HI FBS and 1% Penicillin-streptomycin. Cells were extracted from 3D collagen hydrogels using chloroform and Trizol. RNA was extracted from the cells using Qiagen microRNA RNeasy kit. RNA was quantified using a Nanodrop. RNA was converted to cDNA using the Applied Biosystems^™^ High-Capacity cDNA Reverse Transcription Kit. Human integrin primers were obtained from IDT Inc using the custom DNA oligos. Human primer sequences were found on Origene:

ITGA5 human Forward 5’ – GCCGATTCACATCGCTCTCAAC - 3’
ITGA5 human Reverse 5’ – GTCTTCTCCACAGTCCAGCAAG - 3’
ITGAV human Forward 5’ – AGGAGAAGGTGCCTACGAAGCT - 3’
ITGAV human Reverse 5’ – GCACAGGAAAGTCTTGCTAAGGC - 3’
ITGB1 human Forward 5’ – GGATTCTCCAGAAGGTGGTTTCG - 3’
ITGB1 human Reverse 5’ – TGCCACCAAGTTTCCCATCTCC - 3’
ITGA6 human Forward 5’ – CGAAACCAAGGTTCTGAGCCCA - 3’
ITGA6 human Reverse 5’ – CTTGGATCTCCACTGAGGCAGT - 3’
ITGB4 human Forward 5’ –AGGATGACGACGAGAAGCAGCT - 3’
ITGB4 human Reverse 5’ –ACCGAGAACTCAGGCTGCTCAA - 3’
ITGA2 human Forward 5’ – TTGCGTGTGGACATCAGTCTGG - 3’
ITGA2 human Reverse 5’ – GCTGGTATTTGTCGGACATCTAG - 3’
18S human Forward 5’ –GCAATTATTCCCCATGAACG - 3’
18S human Reverse 5’ –GGGACTTAATCAACGCAAGC - 3’

Human cDNA was amplified using SYBR Green PCR Master Mix (Applied Biosystems) on a Biorad CFX96. TaqMan probes were obtained for the mouse integrin panel:

ITGB1 –Mm01253230_m1, no.: PN4453320, lot no.: P200719-002 A08
ITGB4 –Mm01266840_m1, no.: PN4448892, lot no.: P220809- 000 E04
ITGA2 –Mm00434371_m1, no.: PN4453320, lot no.: P200430-005 A02
ITGA6 –Mm00434375_m1, no.: PN4453320, lot no.: P210328-001 D09
GAPDH-mouse, ref no.: 4352661, lot no.: 1603045.

Mouse cDNA was amplified using TaqMan master mix on a QuantStudio^™^ 3 System.

### Statistical analysis

Data is represented as mean ± SEM. A minimum of 3 independent replicates were performed for each experiment with technical replicates. For studies utilizing tumor organoids, a different mouse model was used for each independent experiment with a minimum of 3 mice used per experiment. We used only female mice since about 99% of breast cancer cases occur in females [42]. Statistical analysis was performed (GraphPad PRISM) using unpaired Student’s t-test, considering p<0.05 as statistically significant.

## Supporting information

S1 Fig ITGβ1 ubiquitous on all cells within tumor organoid. Representative immunofluorescent staining demonstrated ITGβ1 was expressed on leader and follower cells within a tumor organoid in a similar manner.
(TIF)

## References

[1] Nguyen-Ngoc KV, Cheung KJ, Brenot A, Shamir ER, Gray RS, Hines WC, et al. ECM microenvironment regulates collective migration and local dissemination in normal and malignant mammary epithelium. Proc Natl Acad Sci. 2012 Sep 25;109(39):E2595–604. doi: 10.1073/pnas.1212834109 22923691 PMC3465416

[2] Giaquinto AN, Sung H, Miller KD, Kramer JL, Newman LA, Minihan A, et al. Breast Cancer Statistics, 2022. CA Cancer J Clin. 2022;72(6):524–41. doi: 10.3322/caac.21754 36190501

[3] Lu J, Steeg PS, Price JE, Krishnamurthy S, Mani SA, Reuben J, et al. Breast Cancer Metastasis: Challenges and Opportunities. Cancer Res. 2009 Jun 15;69(12):4951–3. doi: 10.1158/0008-5472.CAN-09-0099 19470768

[4] Weigelt B, Peterse JL, van’t Veer LJ. Breast cancer metastasis: markers and models. Nat Rev Cancer. 2005 Aug;5(8):591–602. doi: 10.1038/nrc1670 16056258

[5] Mayor R, Etienne-Manneville S. The front and rear of collective cell migration. Nat Rev Mol Cell Biol. 2016 Feb;17(2):97–109. doi: 10.1038/nrm.2015.14 26726037

[6] Friedl P, Locker J, Sahai E, Segall JE. Classifying collective cancer cell invasion. Nat Cell Biol. 2012 Aug;14(8):777–83. doi: 10.1038/ncb2548 22854810

[7] Cheung KJ, Gabrielson E, Werb Z, Ewald AJ. Collective Invasion in Breast Cancer Requires a Conserved Basal Epithelial Program. Cell. 2013 Dec 19;155(7):1639–51. doi: 10.1016/j.cell.2013.11.029 24332913 PMC3941206

[8] Nathanson SD, Detmar M, Padera TP, Yates LR, Welch DR, Beadnell TC, et al. Mechanisms of breast cancer metastasis. Clin Exp Metastasis. 2022 Feb 1;39(1):117–37. doi: 10.1007/s10585-021-10090-2 33950409 PMC8568733

[9] Cheung KJ, Padmanaban V, Silvestri V, Schipper K, Cohen JD, Fairchild AN, et al. Polyclonal breast cancer metastases arise from collective dissemination of keratin 14-expressing tumor cell clusters. Proc Natl Acad Sci U S A. 2016 Feb 16;113(7):E854–863. doi: 10.1073/pnas.1508541113 26831077 PMC4763783

[10] Hwang PY, Brenot A, King AC, Longmore GD, George SC. Randomly Distributed K14+ Breast Tumor Cells Polarize to the Leading Edge and Guide Collective Migration in Response to Chemical and Mechanical Environmental Cues. Cancer Res. 2019 Apr 15;79(8):1899–912. doi: 10.1158/0008-5472.CAN-18-2828 30862718 PMC6467777

[11] Conklin MW, Eickhoff JC, Riching KM, Pehlke CA, Eliceiri KW, Provenzano PP, et al. Aligned Collagen Is a Prognostic Signature for Survival in Human Breast Carcinoma. Am J Pathol. 2011 Mar 1;178(3):1221–32. doi: 10.1016/j.ajpath.2010.11.076 21356373 PMC3070581

[12] Ray A, Slama ZM, Morford RK, Madden SA, Provenzano PP. Enhanced Directional Migration of Cancer Stem Cells in 3D Aligned Collagen Matrices. Biophys J. 2017 Mar 14;112(5):1023–36. doi: 10.1016/j.bpj.2017.01.007 28297639 PMC5355487

[13] Szulczewski JM, Inman DR, Proestaki M, Notbohm J, Burkel BM, Ponik SM. Directional Cues in the Tumor Microenvironment due to Cell Contraction against Aligned Collagen Fibers. Acta Biomater. 2021 May 14;129:96–109. doi: 10.1016/j.actbio.2021.04.053 33965625 PMC8848478

[14] Wang WY, Pearson AT, Kutys ML, Choi CK, Wozniak MA, Baker BM, et al. Extracellular matrix alignment dictates the organization of focal adhesions and directs uniaxial cell migration. APL Bioeng. 2018 Dec 1;2(4):046107. doi: 10.1063/1.5052239 31069329 PMC6481732

[15] Bays JL, DeMali KA. Vinculin in cell–cell and cell–matrix adhesions. Cell Mol Life Sci. 2017;74(16):2999–3009. doi: 10.1007/s00018-017-2511-3 28401269 PMC5501900

[16] Chen B jun, Tang Y jie, Tang Y ling, Liang X hua. What makes cells move: Requirements and obstacles for leader cells in collective invasion. Exp Cell Res. 2019 Sep 15;382(2):111481. doi: 10.1016/j.yexcr.2019.06.026 31247191

[17] Hwang PY, Mathur J, Cao Y, Almeida J, Ye J, Morikis V, et al. A Cdh3-β-catenin-laminin signaling axis in a subset of breast tumor leader cells control leader cell polarization and directional collective migration. Dev Cell. 2023 Jan 9;58(1):34–50.e9. 36626870 10.1016/j.devcel.2022.12.005 PMC10010282

[18] Rahman A, Carey SP, Kraning-Rush CM, Goldblatt ZE, Bordeleau F, Lampi MC, et al. Vinculin regulates directionality and cell polarity in two- and three-dimensional matrix and three-dimensional microtrack migration. Mol Biol Cell. 2016 May;27(9):1431–41. 26960796 10.1091/mbc.E15-06-0432 PMC4850031

[19] Rothenberg KE, Scott DW, Christoforou N, Hoffman BD. Vinculin Force-Sensitive Dynamics at Focal Adhesions Enable Effective Directed Cell Migration. Biophys J. 2018 Apr 10;114(7):1680–94. doi: 10.1016/j.bpj.2018.02.019 29642037 PMC5954296

[20] Thievessen I, Fakhri N, Steinwachs J, Kraus V, McIsaac RS, Gao L, et al. Vinculin is required for cell polarization, migration, and extracellular matrix remodeling in 3D collagen. FASEB J. 2015 Nov;29(11):4555–67. doi: 10.1096/fj.14-268235 26195589 PMC4608908

[21] Dumbauld DW, Lee TT, Singh A, Scrimgeour J, Gersbach CA, Zamir EA, et al. How vinculin regulates force transmission. Proc Natl Acad Sci. 2013 Jun 11;110(24):9788–93. doi: 10.1073/pnas.1216209110 23716647 PMC3683711

[22] Kim ES, Kim SY, Koh M, Lee HM, Kim K, Jung J, et al. C-reactive protein binds to integrin α2 and Fcγ receptor I, leading to breast cell adhesion and breast cancer progression. Oncogene. 2018 Jan;37(1):28–38. 28846105 10.1038/onc.2017.298

[23] Matsumoto Y, Kage H, Morota M, Zokumasu K, Ando T, Maemura K, et al. Integrin alpha 2 is associated with tumor progression and postoperative recurrence in non-small cell lung cancer. Jpn J Clin Oncol. 2023 Jan 6;53(1):63–73. doi: 10.1093/jjco/hyac148 36151049

[24] Padmanaban V, Grasset EM, Neumann NM, Fraser AK, Henriet E, Matsui W, et al. Organotypic culture assays for murine and human primary and metastatic-site tumors. Nat Protoc. 2020 Aug;15(8):2413–42. doi: 10.1038/s41596-020-0335-3 32690957 PMC8202162

[25] Taufalele PV, VanderBurgh JA, Muñoz A, Zanotelli MR, Reinhart-King CA. Fiber alignment drives changes in architectural and mechanical features in collagen matrices. PLOS ONE. 2019 May 15;14(5):e0216537. doi: 10.1371/journal.pone.0216537 31091287 PMC6519824

[26] Griffin BP, Largaespada CJ, Rinaldi NA, Lemmon CA. A novel method for quantifying traction forces on hexagonal micropatterned protein features on deformable poly-dimethyl siloxane sheets. MethodsX. 2019 Jan 1;6:1343–52. doi: 10.1016/j.mex.2019.05.011 31417850 PMC6690417

[27] Bejar-Padilla V, Cabe JI, Lopez S, Narayanan V, Mezher M, Maruthamuthu V, et al. α–Catenin-dependent vinculin recruitment to adherens junctions is antagonistic to focal adhesions. Mol Biol Cell. 2022 Sep 15;33(11):ar93. 35921161 10.1091/mbc.E22-02-0071 PMC9582796

[28] van der Stoel M, Schimmel L, Nawaz K, van Stalborch AM, de Haan A, Klaus-Bergmann A, et al. DLC1 is a direct target of activated YAP/TAZ that drives collective migration and sprouting angiogenesis. J Cell Sci. 2020 Feb 12;133(3):jcs239947. doi: 10.1242/jcs.239947 31964713

[29] Joosse SA, Hannemann J, Spötter J, Bauche A, Andreas A, Müller V, et al. Changes in Keratin Expression during Metastatic Progression of Breast Cancer: Impact on the Detection of Circulating Tumor Cells. Clin Cancer Res. 2012 Feb 14;18(4):993–1003. doi: 10.1158/1078-0432.CCR-11-2100 22228641

[30] Figueiredo J, Ferreira RM, Xu H, Gonçalves M, Barros-Carvalho A, Cravo J, et al. Integrin β1 orchestrates the abnormal cell-matrix attachment and invasive behaviour of E-cadherin dysfunctional cells. Gastric Cancer. 2022 Jan 1;25(1):124–37. 34486077 10.1007/s10120-021-01239-9 PMC8732838

[31] Yamaguchi N, Mizutani T, Kawabata K, Haga H. Leader cells regulate collective cell migration via Rac activation in the downstream signaling of integrin β1 and PI3K. Sci Rep. 2015 Jan 7;5(1):7656. 25563751 10.1038/srep07656 PMC5379035

[32] Sharma P, Ng C, Jana A, Padhi A, Szymanski P, Lee JSH, et al. Aligned fibers direct collective cell migration to engineer closing and nonclosing wound gaps. Mol Biol Cell. 2017 Sep 15;28(19):2579–88. doi: 10.1091/mbc.E17-05-0305 28747440 PMC5597329

[33] Su CY, Burchett A, Dunworth M, Choi JS, Ewald AJ, Ahn EH, et al. Engineering a 3D collective cancer invasion model with control over collagen fiber alignment. Biomaterials. 2021 Aug 1;275:120922. doi: 10.1016/j.biomaterials.2021.120922 34126408 PMC8450056

[34] Riching KM, Cox BL, Salick MR, Pehlke C, Riching AS, Ponik SM, et al. 3D Collagen Alignment Limits Protrusions to Enhance Breast Cancer Cell Persistence. Biophys J. 2014 Dec 2;107(11):2546–58. doi: 10.1016/j.bpj.2014.10.035 25468334 PMC4255204

[35] Adorno-Cruz V, Liu H. Regulation and functions of integrin α2 in cell adhesion and disease. Genes Dis. 2018 Dec 31;6(1):16–24. 30906828 10.1016/j.gendis.2018.12.003 PMC6411621

[36] Brakebusch C, Fässler R. The integrin–actin connection, an eternal love affair. EMBO J. 2003 May 15;22(10):2324–33. doi: 10.1093/emboj/cdg245 12743027 PMC156003

[37] Liu Y, Keikhosravi A, Mehta GS, Drifka CR, Eliceiri KW. Methods for Quantifying Fibrillar Collagen Alignment. In: Rittié L, editor. Fibrosis: Methods and Protocols [Internet]. New York, NY: Springer; 2017 [cited 2020 Nov 18]. p. 429–51. (Methods in Molecular Biology). 10.1007/978-1-4939-7113-8_28 PMC6343484 28836218

[38] Barcus CE, O’Leary KA, Brockman JL, Rugowski DE, Liu Y, Garcia N, et al. Elevated collagen-I augments tumor progressive signals, intravasation and metastasis of prolactin-induced estrogen receptor alpha positive mammary tumor cells. Breast Cancer Res. 2017 Jan 19;19(1):9. doi: 10.1186/s13058-017-0801-1 28103936 PMC5244528

[39] Liu Y, Keikhosravi A, Pehlke CA, Bredfeldt JS, Dutson M, Liu H, et al. Fibrillar Collagen Quantification With Curvelet Transform Based Computational Methods. Front Bioeng Biotechnol [Internet]. 2020 [cited 2023 Nov 12];8. Available from: https://www.frontiersin.org/articles/10.3389/fbioe.2020.00198 32373594 10.3389/fbioe.2020.00198 PMC7186312

[40] Despotović SZ, Milićević ĐN, Krmpot AJ, Pavlović AM, Živanović VD, Krivokapić Z, et al. Altered organization of collagen fibers in the uninvolved human colon mucosa 10 cm and 20 cm away from the malignant tumor. Sci Rep. 2020 Apr 14;10(1):6359. 32286443 10.1038/s41598-020-63368-y PMC7156654

[41] Berginski ME, Gomez SM. The Focal Adhesion Analysis Server: a web tool for analyzing focal adhesion dynamics. F1000Research. 2013 Mar 4;2:68. doi: 10.12688/f1000research.2-68.v1 24358855 PMC3752736

[42] Anderson WF, Jatoi I, Tse J, Rosenberg PS. Male Breast Cancer: A Population-Based Comparison With Female Breast Cancer. J Clin Oncol. 2010 Jan 10;28(2):232–9. doi: 10.1200/JCO.2009.23.8162 19996029 PMC2815713

