# Supplementary figures and images for: Leader cells mechanically respond to aligned collagen architecture to direct collective migration
Source: PLoS One. 2024 Jan 2;19(1):e0296153. doi: 10.1371/journal.pone.0296153 (PMC10760762; doi:10.1371/journal.pone.0296153)

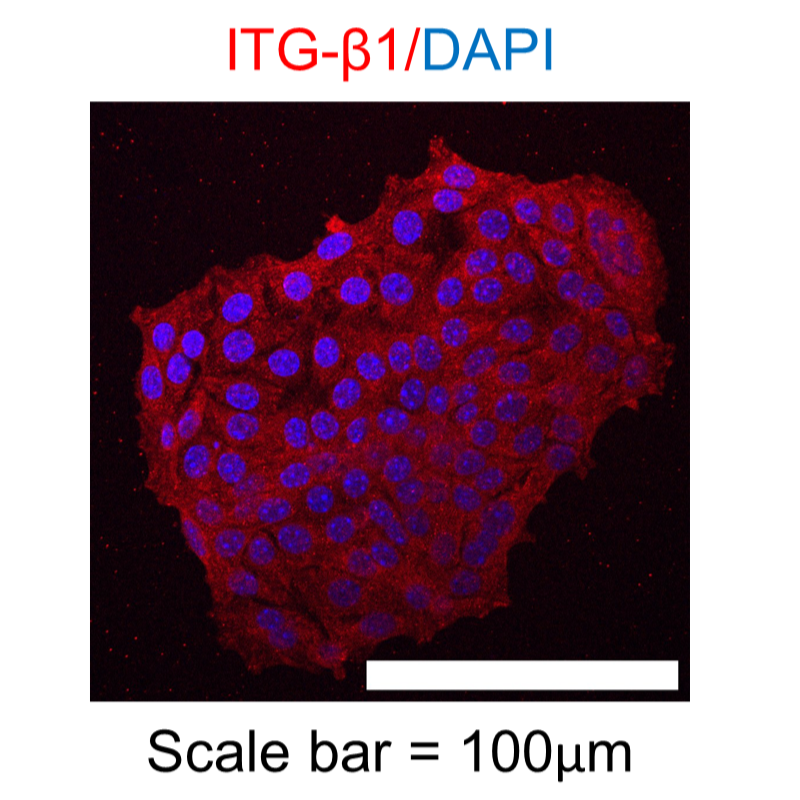

Supplement: S1 Fig — Representative immunofluorescent staining demonstrated ITGβ1 was expressed on leader and follower cells within a tumor organoid in a similar manner. (TIF) [file pone.0296153.s001.TIF]
